# Factors influencing infection in 10 years of battlefield open tibia fractures

**DOI:** 10.1007/s11751-016-0250-x

**Published:** 2016-03-18

**Authors:** J. G. Penn-Barwell, P. M. Bennett, D. E. Mortiboy, C. A. Fries, A. F. G. Groom, I. D. Sargeant

**Affiliations:** National Institute of Health Research, Surgical Reconstruction and Microbiology Research Centre (NIHR SRMRC), Birmingham, UK; Institute of Naval Medicine, Crescent Drive, Gosport, PO12 2DL UK; University Hospital Birmingham, Birmingham, UK; Limb Reconstruction Unit, King’s College Hospital, London, UK

**Keywords:** Combat, War, Injuries and wounds, Open fracture, Tibia, Infection, Limb salvage, Military

## Abstract

The aim of this study was to characterise severe open tibial shaft fractures sustained by the UK military personnel over 10 years of combat in Iraq and Afghanistan. The UK military Joint Theatre Trauma Registry was searched for all such injuries, and clinical records were reviewed for all patients. One hundred Gustilo–Anderson III tibia fractures in 89 patients were identified in the 10 year study period; the majority sustained injuries through explosive weapons (63, 68 %) with the remainder being injured from gunshot wounds. Three fractures were not followed up for 12 months and were therefore excluded. Twenty-two (23 %) of the remaining 97 tibial fractures were complicated by infection, with *S. aureus* being the causative agent in 13/22 infected fractures (59 %). Neither injury severity, mechanism, the use of an external fixator, the need for vascularised tissue transfer nor smoking status was associated with subsequent infection. Bone loss was significantly associated with subsequent infection (*p* < 0.0001, Fisher’s exact test). This study presents 10 years of open tibial fractures sustained in Iraq and Afghanistan. Most infection in combat open tibia fractures is caused by familiar organisms, i.e. *S. aureus*. While the overall severity of a casualty’s injuries was not associated with infection, the degree of bone loss from the fracture was.

## Introduction

As in previous wars, the extremities were the most frequently injured body region during the conflicts of Iraq and Afghanistan [1–3]. Wounds resulting from high-energy military weapons are typically complex and heavily contaminated [4].

Open tibia fractures remain a considerable challenge to the orthopaedic surgeon. Compared to open fractures of other long bones, they have the highest rate of infection [5, 6], likely due to the tibia’s limited soft tissue envelope. Battlefield open tibia fractures have a reported infection rate of 20–30 %, leading to poor outcomes and late amputation [7, 8].

This study aims to expand a previously published 4-year case series [8]. Where the previous study considered infection as a variable affecting outcome, this paper examines deep infection as the outcome and will consider variables contributing to its development.

## Methods

This study was registered with, and approved by, Joint Medical Command. The UK Military Joint Theatre Trauma Registry (JTTR) captures data on all trauma cases admitted to deployed UK military medical facilities who are subsequently repatriated for treatment [9]. The JTTR was searched for codes encompassing bony injuries of the knee, tibia or ankle, sustained between the invasion of Iraq in 2003 and 31 December 2012. The clinical records, X-rays and microbiological results of these cases were reviewed. The following were excluded: cases not involving diaphyseal tibia fractures (AO/Muller type 42); cases other than those graded by the operating surgeon as grade III in the Gustilo–Anderson (GA) classification [10]; and patients who underwent a primary amputation within the first three surgical episodes.

Data were gathered on demographics, injury and surgical management. For the purpose of this study, an injury was considered infected if the infective episode required surgical treatment. A consultant microbiologist analysed all microbiological results and determined the causative micro-organism.

The influence of the following factors on infection was examined:

1. New Injury Severity Score (NISS).
2. Mechanism of injury, i.e. explosive versus gunshot injury.
3. Bone loss [11] (Table 1).

**Table 1:** Bone loss grading system [8]

Grade	Definition
0	*None*
1	*Minimal*, some bone loss but less that 1 cm longitudinally around at least 50 % of the circumference of the shaft but with some cortical contact
2	*Moderate*, bone loss between 1 and 2 cm around at least 50 % of the circumference of the shaft but with some cortical contact
3	*Severe*, bone loss >2 cm around at least 50 % of the circumference of the shaft but with some cortical contact
4	Segmental bone loss
4. Requirement for vascularised tissue transfer (both local and free flaps).
5. Initial stabilisation with an external fixator.
6. Smoking during initial treatment.

### Analysis

Descriptive data are given as medians with inter-quartile range (IQR). Continuous data (i.e. NISS) were analysed by Mann–Whitney analysis. Dichotomous variables were analysed with Fisher’s exact test. In interpreting the levels of statistical significance, a Bonferroni correction was applied to avoid the increased risk of a Type 1 error inherent in multiple comparisons; hence, significance was set at *p* = 0.0083.

## Results

**Fig. 1:**
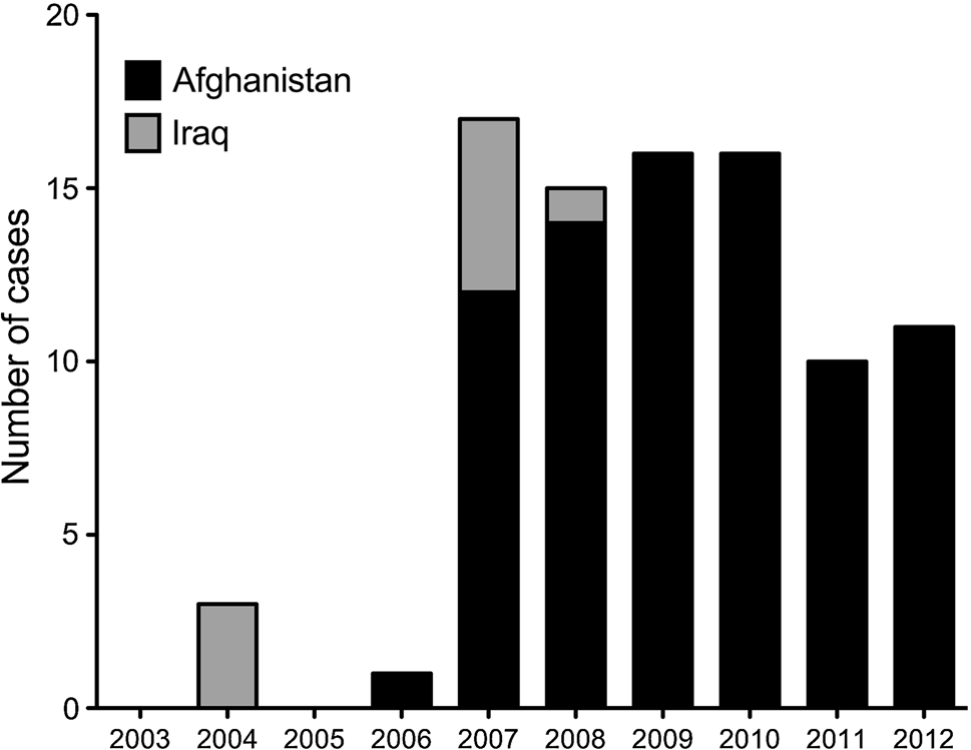
Temporal distribution of casualties over the decade of the study period country of injury shown

The JTTR search identified 445 patients with bony injury affecting the knee, tibia or ankle. Following case note and X-ray review, 353 cases were excluded according to the pre-defined criteria. Eighty-nine patients with 100 severe open tibial fractures were therefore eligible for inclusion (eleven patients with bilateral fractures). The median age was 25 years (IQR 21.3–29.0, mean = 26.0, SD = 5.1). Nine patients were wounded while on service in Iraq with the remainder injured in Afghanistan. The temporal distribution of casualties over the decade is shown in Fig. 1 with the peak occurring in 2007.

Arterial repair was required in seven (7 %) cases, and there was partial or total nerve transection in 13 cases.

Three patients were not followed up for a minimum of 12 months and were therefore excluded from the outcome analysis. In the remaining 97 fractures, the most common technique for definitive treatment was intra-medullary nailing, which was performed in 42 tibial fractures: other fixation methods are given in Table 2. The median delay to definitive fixation was 3 days (IQR 2–4), with no difference in cases that subsequently became infected (*p* = 0.1141). Limb salvage failed in 11 patients, and an amputation was performed; in three cases, this was due to ongoing infection.

**Table 2:** Definitive fracture fixation techniques

Management	*n*	Time to definitive fixation/median days (IQR)
IM nails	42	3 (2–4)
Plates/screws	30	3 (3–5)
External fixation	7	2 (1–2)
Frame	6	52 (33–81)
ORIF with external fixation	5	3 (2–8)
Conservative	9	–

Initial definitive fixation was subsequently revised in 33 cases (34 %), 12 of which were revised to a circular frame with union eventually achieved.

Twenty-two of the 97 cases were complicated by infection. The most common causative micro-organism was *S. aureus* with further detail given in Table 3.

**Table 3:** Causative micro-organisms in the 22 cases who required surgical treatment of infection

Organism	*n*	
*S. Aureus*	13	(1 MRSA)
*Acinetobacter*	3	
*Pseudomonas Sp.*	2	
*Coag. Neg. Staph*	1	
*Enterobacter Sp.*	2	
Unknown	1	

### Injury severity

**Fig. 2:**
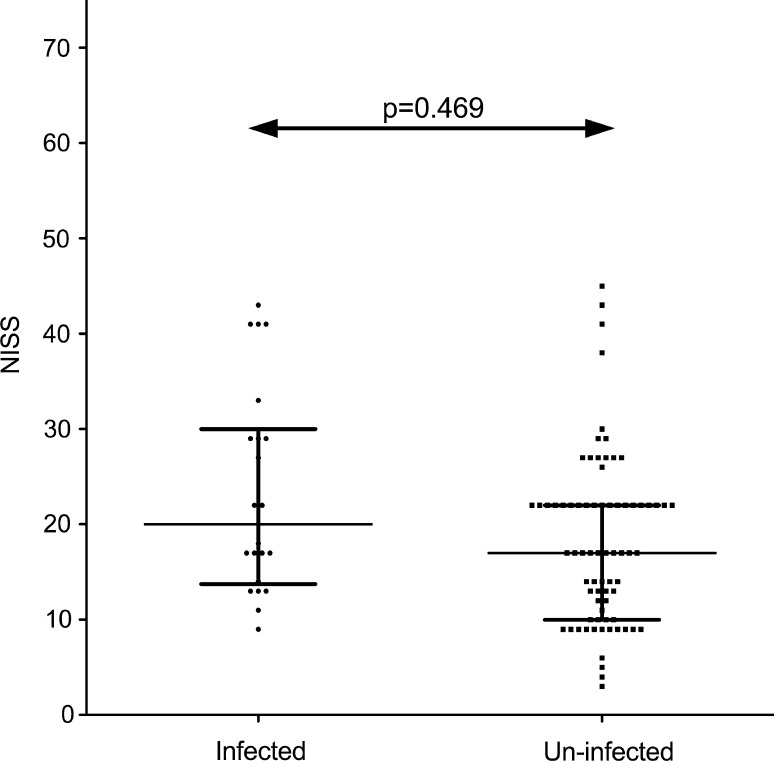
Scatter plot showing the New Injury Severity Score by infected and uninfected groups. The *horizontal lines* denote the median and the inter-quartile range

The median NISS overall was 17 (IQR = 12–22). In the 22 infected cases, the median NISS was 20 (IQR = 15–29) and 17 (IQR = 11–22), in the 75 uninfected fractures (*p* = 0.0469, Mann–Whitney) as shown in Fig. 2. This difference was not regarded as statistically significant due to the Bonferroni correction that was applied.

### Mechanism of injury

The majority of the patients in this series sustained injuries through explosive weapons (72 %) with the remainder being injured from gunshot wounds (GSW). The proportion of injury types was statistically similar in both the infected cohort (GSW = 5/22) and uninfected cohort (GSW = 18/75) (*p* = 0.6014, Fisher’s exact test).

### Bone loss

**Fig. 3:**
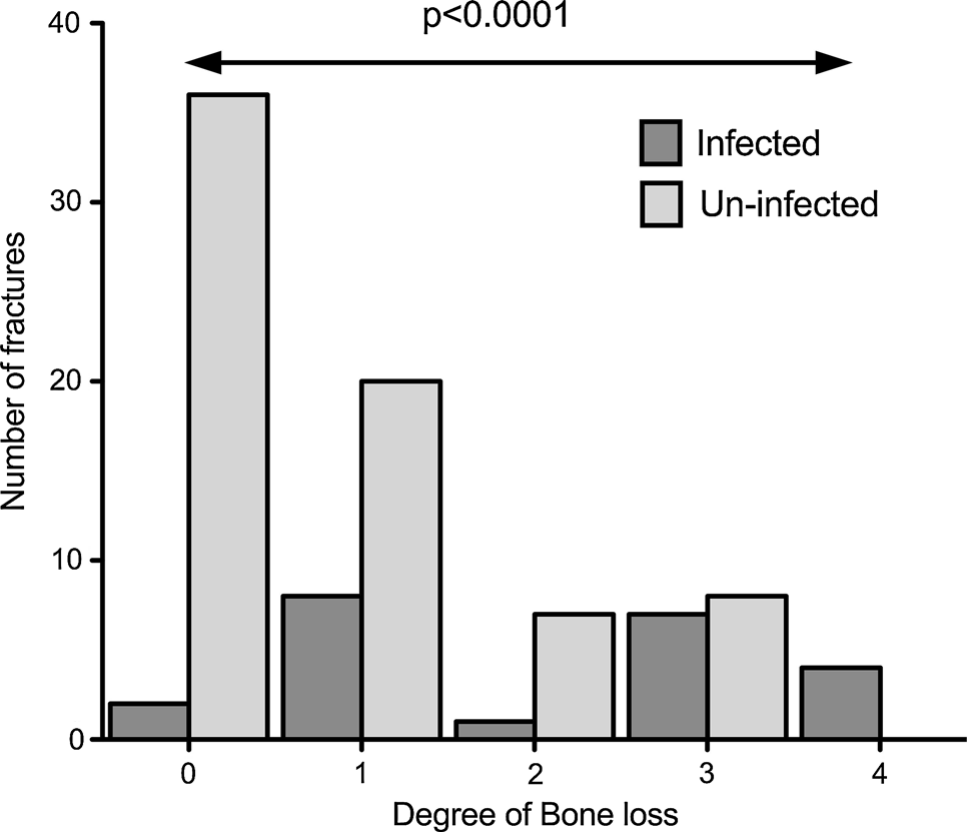
*Bar chart* showing bone loss and infection

The degree of bone loss after debridement is shown in Fig. 3. There were four cases of segmental bone loss in the infected cohort and none in the uninfected group. Bone loss was significantly associated with subsequent infection (*p* < 0.0001, Fisher’s exact test).

### Vascularised tissue transfer

Approximately a third of injuries (31/97) required vascularised tissue transfer, i.e. both local and free flaps: 12 of these subsequently became infected (*p* = 0.0177, Fisher’s exact test). This association was not significant with the application of the Bonferroni correction.

### External fixation

Sixty-one fractures were initially managed with an external fixator (61/97, 63 %). Eighteen of the 22 cases which subsequently developed infection and 43 of the 75 uninfected cases were initially stabilised with an external fixator (*p* = 0.0443, Fisher’s exact test). This association was not significant with the application of the Bonferroni correction.

In 46 cases, external fixation was converted to internal fixation. The median delay until this occurred was 3 days (IQR 2–5). Fourteen of those converted from external fixator to internal fixation developed infection; 32 did not. This was not statistically significant (*p* = 0.09).

### Smoking

Reliable data on smoking status were available for 69 % of fracture cases. Five of 17 infected cases with a recorded smoking status smoked while 18 of 50 uninfected cases did so. This difference was not significant (*p* = 0.7704, Fisher’s exact test).

## Discussion

This study examines the variables associated with the development of deep infection in 10 years of severe open tibial fractures from Iraq and Afghanistan, with an overall infection rate of 23 %. *S. aureus* was the commonest causative organism of deep infection in this cohort. The degree of bone loss was the only variable examined associated with an increased risk of subsequent infection.

### Microbiology

Multiple tissue samples rather than wound swabs should be taken at each surgical episode. A clinical microbiologist should be closely involved in the interpretation of any positive cultures in a multidisciplinary context. This interpretation should consider in vitro results as well as the appearances and behaviour of wounds as they are all at least contaminated and frequently colonised with multiple micro-organisms. However, in our experience, a single micro-organism typically predominates in clinically significant infection, possibly due to the background of extensive antibiotic use in these patients. It is arguably an oversimplification to focus on a single organism in every case, but the authors regard this as a pragmatic approach in the clinical setting.

Sub-clinical infection with low-pathogenic organisms is always suspected in cases of non-union. The authors recommend that these samples are processed by inoculation on to agar plates (primary culture) as well as an enrichment broth for extended culture. The role of molecular methods in diagnosing infection has yet to be determined.

### Infection

Defining infection following severe musculoskeletal trauma is challenging. In this study, a clear definition of infection was used, i.e. infection requiring surgical treatment. This might underestimate the presence of superficial infection, but superficial infections would be difficult to define since all battlefield wounds lie somewhere on the spectrum of contamination–colonisation–infection.

All open fractures were treated aggressively with antibiotics: current UK military practice is to treat open fractures with 1.2 g of intravenous co-amoxiclav every 8 h. Patients showing signs of wound sepsis have their antibiotic therapy tailored to microbiological results. In patients without definitive microbiological results, clindamycin is often given in addition to co-amoxiclav, under the guidance of a consultant microbiologist at the MDT meeting. Patients injured in aquatic environments such as irrigation channels were also treated with ciprofloxacin to cover for atypical gram negatives, e.g. *Aeromonas hydrophilia* [6].

Antibiotics were started as soon after injury as possible and continued typically for longer than the 72 h recommended by the BAPRAS-BOA standards [12]. Prolonged antibiotic therapy is often used given the heavy contamination seen in these injuries despite the recognised lack of evidence for this strategy [5, 13].

Battlefield open fractures are typically heavily contaminated with soil, weapon fragments, clothing and even tissue from other casualties. Despite this, 77 % of injuries in this series were not complicated by infection. This is comparable to the findings of a similar case series of US military casualties with open tibia diaphysis fractures, which reported an infection-free rate of 73 % using the same definition as this study [7].

The finding that *S. aureus* is the most common pathogenic micro-organism is similar to the findings of other investigators [7]. The authors speculate that *S. aureus* might survive in open fractures due to its ability to form a biofilm on bone or metal or persist intra-cellularly in osteoblasts [14, 15]. Future research should be aimed at addressing *S. aureus* persisting using these mechanisms.

### Surgical strategy

The strategies used in our institution to prevent infection in patients with open fractures aim to promote a healthy wound bed and stable soft tissue envelope. Central to this effort is meticulous surgical debridement with removal of contamination and necrotic tissue. This is often performed repeatedly due to wound evolution, as vulnerable but seemingly viable tissue can become necrotic. This is particularly evident with blast injuries. Wounds are irrigated with saline due to the damaging effects of antiseptic solutions on vulnerable host tissue [16, 17].

Skeletal stability is the key to protecting the soft tissue, and early definitive fixation is employed as soon as wound evolution has ceased. We regard this as important in a successful host response to infection. Many of these patients are pyrexial for many weeks despite empirical or directed antibiotic therapy. The underlying cause of this is frequently not determined, but is not thought to be a result of infection. Delaying fracture fixation until patients are apyrexial might result in long delays until fixation. The timing of wound closure or coverage was dictated by the surgeon’s judgement of the health of the wound, and negative wound cultures were not a prerequisite for this. It is recognised that wounds are likely to be colonised with bacteria, but this does not equate to acute infection.

Ideally wound closure or coverage is performed at the same time as fixation in accordance with the BOA/BAPRAS standards. However, combat wounds can be extensive and involve a considerable proportion of the limb. Frequently the depths of the wound in the immediate fracture zone become visibly clean and viable before the totality of the wound has closed or been covered. In these complex wounds it is not uncommon for the metalwork to be covered primarily or with vascularised tissue transfers, while more distant portions of the same wound remain open, with topical negative pressure dressings in place. For this reason, in many wounds it is impossible to define a specific point that the wound is closed or covered.

Skeletal fixation is often undertaken for bony stability rather than to allow early full weight bearing. This strategy is used in patients not capable of early weight bearing due to coexisting injuries of limbs, trunk and CNS. The principle of minimising the quantity of fixation implants used not only reduces the surgical insult but maximises the number of fractures that can be stabilised in a short anaesthetic period in a sick, febrile patient.

### Interpretation of results

Injury severity can be measured either generally, i.e. with a overall injury severity score such as NISS, or locally by examining soft tissue and bone loss. Like other investigators, we found that overall injury severity was not associated with risk of infection [18].

In their seminal description of over a 1000 open fractures, Gustilo and Anderson developed a classification system based predominantly on the extent of soft tissue damage. They described the link between the severity of the local injury and the risk of infection [10]. In this study, there was no direct attempt to further subdivide the fractures into Gustilo–Anderson grade III groups A, B and C [19]. However, the use of vascularised tissue transfer, either local or free flaps, can be regarded as synonymous with grade IIIB injuries. Local injury severity with respect to soft tissue damage and the requirement for vascularised soft tissue transfer was not found to be associated with infection.

Local injury severity determined by bone loss was strongly associated with infection in this study. It is reasonable to speculate that significant bone loss is a good surrogate marker for limb injury severity, i.e. extensive, high-energy damage to both bone and the soft tissue envelope.

External fixation is widely regarded as a safe technique for temporarily stabilising open fractures prior to definitive internal fixation [20]. This is borne out by our findings and by the results of a study of 55 open tibia fractures in US military personnel treated in this manner [21]. This study did not demonstrate an association between the temporary stabilisation of fractures with external fixators and the subsequent development of infection.

Smoking is recognised as being associated with wound healing problems in orthopaedic surgery [22, 23]. However, this was not supported by the findings of this study. The authors speculate that the vasculature of the relatively young smokers in this study has not yet been damaged by the effects of tobacco.

### Study weaknesses

The authors readily admit that there are weaknesses associated with the design of this study. Firstly, an observational study of this type can only describe association, not causation. Secondly, smoking can be hard to determine from hospital notes; however, records from the Defence Medical Rehabilitation Centre reliably report on tobacco and alcohol use as part of the social assessment of the patient.

Five of the six variables examined in this paper are non-modifiable. Initial fixation with stabilisation prior to internal fixation was the only modifiable variable studied and was not found to be associated with subsequent infection. Though other modifiable risk factors such as limb ischaemic time and transfusion requirement could be examined, the severely injured nature of these casualties would preclude any deviation from established initial treatment algorithms for consideration of the reduction in the risk of later infection. It is also worth noting that it has not been definitively established whether patient-reported outcomes are superior in limb salvage or amputation following severe tibial injury in service personnel [24, 25].

Despite these acknowledged weakness, this study describes all of the severe open tibia shaft fractures sustained by the UK military personnel over a decade of combat in Iraq and Afghanistan. The infection rate is defined as 23 %, and the most common causative micro-organism is confirmed as *S. aureus*. The only factor significantly associated with an increased incidence of infection was found to be the extent of bone loss.
